# Supplementary material for: Knowledge of modifiable cardiovascular diseases risk factors and its primary prevention practices among diabetic patients at Jimma University Medical Centre: A cross-sectional study
Source: PLOS Glob Public Health. 2022 Jul 12;2(7):e0000575. doi: 10.1371/journal.pgph.0000575 (PMC10022065; doi:10.1371/journal.pgph.0000575)
Supplement: Attachment — Submitted filename: Response to reviewers .pdf [file pgph.0000575.s001.pdf]

Author's response to reviewers

Manuscript Title: Knowledge of Modifiable Cardiovascular Diseases Risk Factors and Its Primary Prevention Practices among Diabetic Patients at Jimma University Medical Centre: A cross-sectional study

Authors:

Abdata Workina(abdeta.15@gmail.com)

Asaminew Habtamu

Tujuba Diribsa

Fikadu Abebe

To Editors and Reviewers: Thank you for allowing us to submit a revised draft of our manuscript. We appreciate the time and effort that you and the reviewers have dedicated to providing your valuable feedback on our manuscript. We are grateful to the reviewers for their insightful comments on our paper. We have been able to incorporate changes to reflect the suggestions provided by the reviewers in the response letter, revised manuscript with track changes, and manuscript. We hope it will now be suitable for publication in the **PLOS Global Public Health** journal.

Yours sincerely!

Here is a point-by-point response to the reviewers' comments and the editor's concerns.

Journal Requirements:

1. Please amend your Financial Disclosure statement. If you did not receive any funding for this study, please simply state: "The authors received no specific funding for this work."

   Response: Thank you, we have amended it according to the suggestion (Page- 15)

2. Please update your Competing Interests statement. If you have no competing interests to declare, please state: "The authors have declared that no competing interests exist."

Response: Thank you, we have stated it (Page- 15)

3. In the online submission form, you indicated that “The datasets generated and analyzed during the current study are not publicly available due to these data were used under license for the current study but are available from the corresponding author on reasonable request.”. All PLOS journals now require all data underlying the findings described in their manuscript to be freely available to other researchers, either 1. In a public repository, 2. Within the manuscript itself, or 3. Uploaded as supplementary information. This policy applies to all data except where public deposition would breach compliance with the protocol approved by your research ethics board. If your data cannot be made publicly available for ethical or legal reasons (e.g., public availability would compromise patient privacy), please explain your reasons by return email and your exemption request will be escalated to the editor for approval. Your exemption request will be handled independently and will not hold up the peer review process, but will need to be resolved should your manuscript be accepted for publication. One of the Editorial team will then be in touch if there are any issues.

   Response: The essential data used for the conclusion were available within the manuscript itself.

4. Please ensure that the Title in your manuscript file and the Title provided in your online submission form are the same.

   Response: Thank you, we have checked it.

Comments from Reviewer #1

Comment 1: A good study but the conclusion needs to be re-worded. The second paragraph of the discussion reads "...This study shows that more than half, 198(62.3%), of the study participants had good Knowledge of modifiable CVDs risk factors..."; therefore, the conclusion that "-----The study participants'

knowledge of modifiable cardiovascular disease risk factors was insufficient", does not seem congruent since the majority (over half) of respondents have good knowledge

Response: Thank you; we agree with your comment and we have reworded it both in the conclusion part of the abstract and in the conclusion heading of a manuscript (Page-14). i.e., we have rephrased it to “The study participants' knowledge of modifiable cardiovascular disease risk factors was satisfactory, even though continuous awareness creation is required to lower CVD mortality and morbidity burdens.”

Comment 2: Rephrase the last paragraph of the conclusion (typographical errors too).

Response: Thank you we have corrected it (Page 15, paragraph 5).

Comments/Suggestions from Reviewer #2

1. **Page 4_ Suggestion:** insert the period in which the survey took place, as well as the variables

Response: thank you we have added it (page 4**).**

2. Page 5**-**This study shows that more than half, 198(62.3%), of the study participants had good Knowledge of modifiable CVDs risk factors and in Results, 175(55.0%) of the patients had a good CVD prevention practice, and if 55.5%, that is, more than half of the population studied had good practices, it is risky to state in the conclusion that "practices are insufficient"... Bias!? Suggestion: review!

   Response: thank you, we have addressed the suggestion **(page -15)**

3. Page-6 Suggestion: relocate this paragraph as first.

   Response: We have relocated it.

4. Page 7-Little study time. Why? Suggestion: look for other similar studies to verify the research time issue. Justify!

   Response: The period of data collection was around 7 weeks to gather information from 318 patients out of 1260 diabetic patients. These study participants' size and their availability were shorten the duration of data collection. besides, we searched other similar cross-sectional studies which shows somewhat related in terms of data collection considering size and procedures of sample selection.

5. Page 8- What are the issues? How many?

Response: The sensitive issues we had been intended to encounter before pre-test were behavioural risk factors of cardiovascular disease. However, during pre-test and data collection it was not sensitized by study participants.

6. Page 9-Suggestion: information must be in the Abstract

   Response: thank you. We have included it in the abstract. (Abstract -->result)

7. Page 11-If this study shows that more than half, 198(62.3%), of the study participants had good Knowledge of modifiable CVDs risk factors and 55.5%, that is, more than half of the population studied had good practices, it is risky to state in the conclusion that "practices are insufficient"... Bias!? Suggestion: review!

Response: thank you, we have reviewed and corrected it. (Page 15, conclusion--> paragraph 2)

8. Page 12-Why this value and not 0.5? Suggestion: justify!

Response: Since most researchers recommend using <0.05 to indicate that there is <5% chance of making type one error when rejecting the null hypothesis. That is why we used <0.05 instead of ≤ 0.05.

9. Page15-Suggestion: discuss the results found and compared studies. Discuss the main points justifying and giving perspectives... It lacked to justify the importance and the reason of the study carried out as a whole.

   Response: Thank you, we have tried to address it. **(Discussion section)**

10. Page16- And what is the conclusion of this? Suggestion: develop what has been compared...discuss such facts! Remove data, percentages.

Response: Thank you, we have tried to correct it.

11. Page 17-Suggestion: I would also add the study time as a limiting factor, as well as the calculation of the listed population.

Response: We have added study time as a limiting factor but on the sample size calculation procedure, we had gone through a justifiable scientific formula to calculate the study population.

Thank you!
